# Stress-Coping Strategies and Factors Related Distress among Japanese Physicians

**DOI:** 10.18502/ijph.v49i7.3599

**Published:** 2020-07

**Authors:** Yuichiro OTSUKA, Yoshitaka KANEITA, Osamu ITANI, Sachi NAKAGOME, Maki JIKE, Takashi OHIDA

**Affiliations:** Division of Public Health, Department of Social Medicine, School of Medicine, Nihon University, 30-1 Oyaguchi-kamimachi, Itabashiku, Tokyo, Japan

## Dear Editor-in-Chief

Physicians experience distress in the workplace such as overworking, managerial responsibilities, maintenance of relationships with patients and their families, the residency training system and changes that it involves, and the increasing litigation risk (1). In very stressful situations, implementing an appropriate method of stress coping can prevent stress-related diseases (2).

We aimed to survey the association between distress and stress-coping strategies among Japanese physicians, including work situations. A self-administered questionnaire survey was conducted among the Japan Medical Association members. The randomly selected participants comprised of 6,000 male and 1,500 female physicians.

All survey respondents provided informed consent to participate. The questionnaire comprised questions about the following: 1) basic attributes: gender, age, type of work, institution, and department; 2) work status: working hours, number of holidays, and number of days of on-call/overnight work; 3) sleep situation; 4) experience of medical incidents; 5) awareness of distress; and 6) stress-copings. The following options were provided as stress coping strategies, and the subjects were able to choose any of them (3): 1- Addressing problems; 2- Performing exercise; 3- Enjoying hobbies or relaxing alone; 4- Watching television or listening to the radio; 5- Sharing worries and concerns with family and friends; 6- Giving up on problem-solving; 7- Enduring problems; 8- Thinking positively and muddling through problems; 9- Looking for stimulation and excitement; 10- Drinking alcohol; 11- Smoking; 12- Eating something). Each logistic regression analysis was performed to examine factors associated with distress in physicians and to survey the association between distress and stress-coping strategies. All the analyses were performed using SPSS 17.0 (IBM Corp., Somers, NY, USA). *P*<0.05 were considered statistically significant.

Valid responses were obtained from 5,854 physicians (4,267 male and 1,227 female). The prevalence of distress was 12.4% among males and 12.1% among female physicians. Table 1 shows the factors associated with distress in Japanese physicians identified by simple and multiple logistic regression. Factors associated with distress such as age group, employment status, type of medical institution, working hours, medical incidents, and sleeping hours. The results were similar to previous studies (1, 4). Table 2 shows the association between distress and stress-coping strategies in Japanese physicians. Stress coping strategies such as ‘Enduring problems patiently’ and “Eating something” were positively associated with distress.

**Table 1:** Factors associated with distress in Japanese physician

*** Variable ***	*** Category ***	*** Crude OR ***	*** 95%CI ***	*** AOR ***	*** 95%CI ***
Gender	Male	1.00		1.00	
	Female	0.98	0.81–1.19	1.12	0.89–1.41
				
Age group(yr)	20–39	1.00		1.00	
	40–49	1.48	1.08–2.02	1.64	1.15–2.32
	50–59	1.23	0.91–1.67	1.34	0.94–0.91
	60–69	1.00	0.73–1.39	1.26	0.86–1.84
	70+	0.62	0.44–0.88	0.92	0.58–1.45
Working form	Employers	1.00		1.00	
	Employees	0.95	0.81–1.11	0.65	0.49–0.96
Medical institution	Clinic	1.00		1.00	
	Hospital	1.29	1.10–1.52	1.43	1.07–1.91
	Other	0.71	0.46–1.11	0.86	0.46–1.61
Working hours/day	<6	0.90	0.57–1.41	1.09	0.66–1.80
	≥6, <8	1.00		1.00	
	≥8, <10	1.62	1.19–2.21	1.44	1.03–2.00
	≥10, <12	2.84	2.08–3.89	2.31	1.62–3.27
	≥12	4.70	3.39–6.52	2.90	1.97–4.25
Holidays /month	<4	1.69	1.37–2.09	1.19	0.93–1.50
	≥4, <6	1.00		1.00	
	≥6, <8	0.68	0.52–0.88	0.77	0.58–1.02
	≥8, <10	0.77	0.61–0.98	0.98	0.76–1.28
	≥10	0.52	0.39–0.69	0.80	0.58–1.13
Night shift /month	Never	1.00		1.00	
	Rarely	1.09	0.78–1.52	0.84	0.58–1.21
	Once	1.17	0.85–1.59	0.78	0.55–1.09
	2–3	1.35	1.02–1.79	0.85	0.61–1.18
	4–7	1.28	0.98–1.69	0.80	0.58–1.11
	≥8	2.62	1.96–3.50	1.43	1.02–1.99
Medical incidents /month	Never	1.00		1.00	
	Rarely	1.02	0.81–1.29	0.90	0.70–1.16
	Sometimes	2.02	1.59–2.58	1.66	1.28–2.17
	Often	5.83	3.79–8.97	4.11	2.58–6.54
Sleeping hours/day	<5	3.72	2.63–5.28	2.04	1.36–3.05
	≥5, <6	2.42	1.90–3.08	1.71	1.31–2.24
	≥6, <7	1.49	1.20–1.84	1.26	0.99–1.59
	≥7, <8	1.00		1.00	
	≥8	1.23	0.90–1.69	1.50	1.05–2.16

All the items included in this table were input in the logistic model

**Table 2:** The association between distress and stress-coping strategies in Japanese physician

*** Variable ***	*** Male ***			*** Female ***		
	*** AOR ***	*** 95%CI ***	*** P-value ***	*** AOR ***	*** 95%CI ***	*** P-value ***
Addressing problems	0.94	0.76–1.16	0.548	1.02	0.67–1.55	0.931
Performing exercise	0.79	0.64–0.99	0.036	0.70	0.42–1.19	0.185
Enjoying their hobbies or relaxing alone	0.64	0.52–0.78	<0.001	0.47	0.31–0.72	<0.001
Watching television or listened to the radio	0.98	0.78–1.23	0.844	1.28	0.82–1.98	0.275
Sharing worries and concerns with family and friends	1.38	1.07–1.77	0.012	1.16	0.77–1.76	0.477
Giving up on problem-solving	1.02	0.69–1.51	0.913	1.27	0.63–2.58	0.510
Enduring problems	2.95	2.40–3.62	<0.001	3.59	2.35–5.47	<0.001
Thinking positively and mudding through problems	0.86	0.70–1.06	0.150	0.78	0.51–1.18	0.235
Looking for stimulus and excitement	1.15	0.64–2.09	0.637	4.11	0.95–17.70	0.058
Drinking alcohol	1.19	0.95–1.48	0.127	1.08	0.58–2.00	0.802
Smoking	1.59	1.07–2.35	0.022	0.96	0.22–4.23	0.961
Eating something	1.39	1.03–1.88	0.030	1.46	0.91–2.36	0.117

Adjusted for age group, working form, medical institution, working hours, holidays, night shift, experience of medical incidents, and sleeping hours by the multiple logistic regression analysis

When their job satisfaction is low, males resort to drinking more alcohol (4), whereas females are reportedly more prone to overeating when faced with stress (5). Besides, ‘Enjoying hobbies or relaxing’ and “Physically active and performing exercise” were negatively associated with distress.

These strategies were suggested positive effect on physical and mental health (6). Stress-coping strategies were important in the reduction and increasing distress in Japanese physicians.
